# Hybrid Crude Palm Oil in Brazilian Regions: Evaluation of Knowledge, Perceptions, and Consumption Potential

**DOI:** 10.3390/foods14183242

**Published:** 2025-09-18

**Authors:** Agnes Sophia Braga Alves, Deborah Murowaniecki Otero, Alana Moreira Bispo, Fabiane do Espírito Santo de Jesus, Edilene Ferreira da Silva, Lívia de Matos Santos, Itaciara Larroza Nunes, Maria Cristina Teixeira Cangussu, Cláudio Vaz Di Mambro Ribeiro, Camila Duarte Ferreira Ribeiro

**Affiliations:** 1 Graduate Program in Food Science, School of Pharmacy, Federal University of Bahia, Ondina Campus, Salvador 40170-115, Bahia, Brazil; agnes.braga@ufba.br (A.S.B.A.); deborah.otero@ufba.br (D.M.O.); liviamatos@ufba.br (L.d.M.S.); claudioribeiro@ufba.br (C.V.D.M.R.); 2 Graduate Program in Food, Nutrition, and Health, School of Nutrition, Federal University of Bahia, Canela Campus, Salvador 40110-907, Bahia, Brazilfabianej@ufba.br (F.d.E.S.d.J.); 3 Graduate Program in Food Science, Department of Food Science and Technology, Federal University of Santa Catarina, Admar Gonzaga Road, 1346, Itacorubi, Florianópolis 88034-000, Santa Catarina, Brazil; silva.e.f.s@posgrad.ufsc.br (E.F.d.S.); itaciara.nunes@ufsc.br (I.L.N.); 4 Graduate Program in Dentistry and Health, School of Dentistry, Federal University of Bahia, 62-Canela Campus, Salvador 40110-150, Bahia, Brazil; cangussu@ufba.br; 5 School of Veterinary Medicine and Animal Science, Federal University of Bahia, Av. Adhemar de Barros, 500, Salvador 40170-110, Bahia, Brazil

**Keywords:** *Elaeis guineensis*, *Elaeis oleifera*, hybrid palm oil, palm oil with a higher content of oleic acid, online questionnaire, consumer, Brazil

## Abstract

The hybrid *Elaeis oleifera* × *E. guineensis* crude palm oil (HCPO), when compared to traditional crude palm oil (CPO), *E. guineensis*, presents higher levels of oleic acid, vitamin E, and carotenoids, and agronomic advantages for cultivation. This study aimed to analyze the perception, knowledge, and potential consumption of hybrid crude palm oil in different regions of Brazil. Data collected through an online questionnaire with 16 questions revealed that Brazilian consumers (*n* = 1065) had a limited understanding. Most responses (61.10%) did not accurately define this type of oil. The overall perception of the HCPO was predominantly neutral. Participants from the Southeast and Northeast regions had a more positive perception of the HCPO. The survey indicated that most participants (52.58%) expressed interest in trying HCPO, whereas 39.43% showed interest in buying products containing this oil, suggesting its potential market acceptance in Brazil. Future studies may encourage the popularization of this oil through the characterization, development of food products, and investigation of its health effects.

## 1. Introduction

Crude palm oil derived from the fruit of the African oil palm (*Elaeis guineensis*) is the most widely produced and traded vegetable oil in the world, contributing significantly to food production, industrial applications, and biofuels [1,2]. For the 2024/25 harvest, global production is estimated to be between 79 and 81 Mt, with Southeast Asia, particularly Indonesia, Malaysia, and Thailand, leading both production and exports [2]. Brazil ranks eighth, with a production of approximately 600,000 t, corresponding to 0.8% of the global production, mostly destined for domestic consumption [3]. Pará and Bahia account for most of the production of conventional crude palm oil (CPO) [4].

In Bahia, artisanal and extractive production still persists, albeit in decline, maintaining manual methods that reflect historical traditions and a strong connection to local culture. In contrast, industrial palm oil production in Pará has been expanding, driven by investments in technology and mechanization, with the aim of meeting growing demand of the global market [5]. In Bahia, crude palm oil is widely used in traditional culinary preparations such as acarajé, abará, vatapá, and quiabada [6].

Although highly productive, *Elaeis guineensis* cultivation has limitations, including susceptibility to practices and diseases such as fatal yellowing (FY) or bud rot (BR) in Central and South America, as well as management difficulties due to vertical stem growth [7,8]. To overcome these limitations, the hybrid *Elaeis oleifera* × *E. guineensis* crude palm oil (HCPO) interspecific hybrids, known as the HIE OxG or OxG hybrid, were developed from crosses between *E. oleifera* (native to Central and South America) and *E. guineensis*. This hybrid combines the high productivity of African palm oil with the superior agronomic attributes of American palm oil, such as resistance to FY, a higher unsaturated fatty acid content, and a smaller stem, facilitating management and increasing production longevity [7].

The main producers of HCPO are Colombia (accounting for 12% of the total area cultivated for this oil), Ecuador, and Costa Rica [9]. In Brazil, the first hybrid variety launched by BRS Manicoré, developed by Embrapa in 2010, was a cross between the Caiaué palms of Manicoré origin (Amazon, Brazil) and palms of the La Mé variety (Ivory Coast) [7]. Extended experiments by the Executive Commission of the Cocoa Crop Plan (Ceplac) in Una, Bahia, with a hybrid variety developed by Embrapa Western Amazon (AM) resulted in the production of a HCPO with low acidity and a milder flavor, which may positively impact Bahian cuisine, since the high acidity of CPO is associated with gastrointestinal disorders and consumption limitations [10].

**Figure 1:**
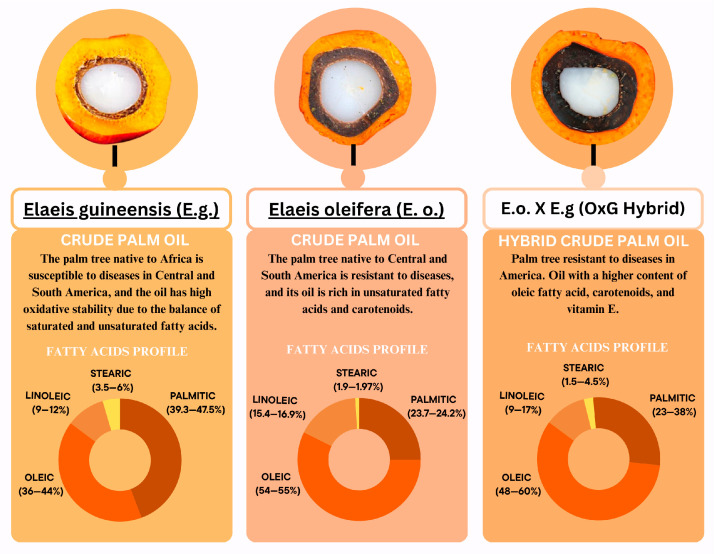
Differences between crude palm oils (*Elaeis guineensis* and *Elaeis oleifera*) and hybrid crude palm oil (*Elaeis guineensis* x *Elaeis oleifera*). Note: [1,7,23,24].

From a chemical-nutritional perspective, HCPO has a higher content of unsaturated fatty acids, particularly oleic acid, and a lower proportion of saturated fatty acids (Figure 1). According to CODEX [1], due to its oleic acid content, it is recognized as the “palm oil with the highest oleic acid content.” Furthermore, it has a high carotenoid content, ranging from 500 to 10,000 µg/g, composed mainly of β-carotene (52–60%) and α-carotene (33–36%) [11]. The tocopherol and tocotrienol content ranges from 562 to 1417 mg/kg, with γ-tocotrienol being the predominant component (406–887 µg/g) [1]. It also contributes to the reduction in inflammatory markers such as IL-6, stimulation of antioxidant enzymes, and cellular protection [11,12].

HCPO is called the “tropical equivalent of olive oil” due to its similar phenolic compound profile and comparable health effects on cardiovascular risk factors [13], as shown in studies by Rodríguez et al. [14], Ojeda et al. [15] and Lucci et al. [16]. However, this equivalence was confirmed by Spreafico et al. [17] and Sales et al. [18], who found that HPO consumption could induce non-alcoholic fatty liver disease (NAFLD) and liver damage in animal models. Furthermore, Gesteiro et al. [19] argued that a direct comparison was invalid because oils have not been tested within the same dietary context, such as the Mediterranean diet.

In a recent study, Villamil et al. [20] demonstrated the functional potential of yogurts with total replacement of milk fat by hybrid palm oil, the participants’ lipid profile improved, showing a significant reduction in total cholesterol (TC) and low-density lipoprotein cholesterol (LDL-C) after 3 months of daily consumption, likely due to the high content of unsaturated fatty acids and vitamin E. Assunção et al. [21] demonstrated that nanoparticles of HCPO encapsulated in jackfruit by-products exhibited superior cellular antioxidant activity compared to free HCPO, while crucially inducing no cytotoxic effects on human intestinal Caco-2 cells, suggesting a promising delivery systems for future HCPO applications. In addition Oliveira et al. [22] demonstrated that HCPO nanoparticles efficiently preserved refrigerated beef burgers and have potential as natural food nanopreservatives.

In this context, HCPO has emerged as a promising alternative for addressing the nutritional, social, environmental, and economic challenges associated with palm oil production chain. In Brazil, where the demand for vegetable oils continues to grow, HCPO can contribute to food and nutritional security and strengthen the agricultural economy. However, knowledge about this hybrid variety remains limited, making it essential to assess consumer perception, knowledge, and consumption potential to guide marketing strategies, increase acceptance, and ensure successful product adoption [25]. Thus, the present study aims to analyze the perceptions, knowledge, and consumption potential of HCPO in different regions of Brazil.

## 2. Methodology

### 2.1. Study Design and Participants

This study employed a cross-sectional design to assess the knowledge, perceptions, and consumption potential of HCPO in various Brazilian regions. A nonprobabilistic convenience sampling method was used to recruit participants. Although the sampling was nonprobabilistic, a reference for the minimum number of participants was estimated as indicative. Based on standard sample size assumptions (95% confidence interval, z = 1.96, *p* = 0.5) and considering the Brazilian adult population in 2022 (*N* = 203,080,756), the minimum required sample would be approximately 385 participants. In this study, however, a total of 1065 participants were obtained, ensuring the same confidence level with an estimated sampling error of 3%. Participants were recruited through social media links such as email and messaging apps (specifically, Instagram v105.0.0.18.119 and WhatsApp v2.22.25.13). The inclusion criteria required participants to be residents of Brazil, aged 18 years or older, have internet access, and agree to the informed consent form (ICF). Individuals who did not sign the ICF or answered the verification question incorrectly at the end of the questionnaire were excluded. Participants’ confidentiality and anonymity were maintained.

### 2.2. Data Collection

Between March and July 2022, data were collected using Google Forms, following approval by the Ethics Committee of the Federal University of Bahia (23 March 2022), Brazil (registration number: 5.306.483; Certificate of Ethical Presentation (CAAE) number: 56357822.1.0000.5023).

Data were collected using a single questionnaire, consisting of two complementary studies. The first published study [25] addressed crude and refined palm oil in Brazilian regions. The second study presented in this manuscript focuses on hybrid crude palm oil.

To ensure data quality, the questionnaire included attention-check items, and validation procedures were applied to exclude incomplete submissions. Mandatory response fields prevented missing data, and all participants’ data were treated with strict confidentiality and anonymity.

An analysis of the target audience’s knowledge, perception, and potential consumption was conducted using both qualitative and quantitative methods, totaling 15 questions distributed as follows: seven general questions regarding socioeconomic aspects; eight questions to assess the knowledge, perception, and potential consumption of hybrid crude palm oil; and one verification question (Supplementary Materials).

**Figure 2:**
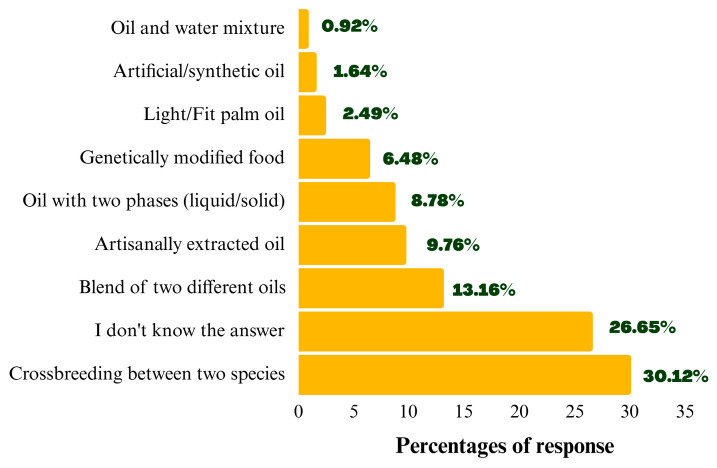
Response Frequency on Knowledge of Hybrid Crude Palm Oil.

A question with ten options was presented to assess knowledge, allowing participants to select multiple options (Figure 2). A five-point Likert scale was used to measure perception and potential consumption (Table 1).

To evaluate the attributes, the following criteria were used: positive perception if it presented at least three of the following factors: healthy, had more nutrients than its parents, had low acidity, did not cause gastrointestinal symptoms, and did not alter the characteristics of traditional Bahian dishes; negative perception if it presented at least three of the following factors: unhealthy, had fewer nutrients than its parents, had high acidity, caused gastrointestinal symptoms, and altered the characteristics of traditional Bahian dishes; and neutral perception if it presented at least three neutral responses regarding the factors mentioned above.

### 2.3. Statistical Analysis

The data are expressed as absolute and relative frequencies and are presented in tables, graphs, and figures. Correspondence analysis was used to identify associations between categories related to the region of residence and questions regarding perceptions and purchase potential. Chi-square analysis was used to compare data on sex, age, household income, and region. The data were statistically evaluated using the STATISTICA software version 7.0 and MINITAB (version 14.0). Additional tables, graphs, and figures were created using Excel and the graphic design website CANVA (Sydney, Australia, 2012).

## 3. Results

### 3.1. Consumer Profile

From an initial pool of 1082 respondents, 17 were excluded because of failed attention checks, resulting in a final analytical sample of 1065 participants. The socioeconomic data presented and discussed by Bispo et al. [25] were synthesized to support an understanding of the study’s results. After applying the inclusion criteria, 1065 responses from the online questionnaire were considered, with most respondents being Brazilians residing in the northeastern region (40.37%) (Table 2). The participants were predominantly women (69.76%), aged 30–39 years (27.51%), and living alone (12.68%). The majority held a postgraduate degree (63%), were employed full-time (61.60%), and had an income exceeding nine times the minimum wage (35.87%).

### 3.2. Knowledge of Hybrid Crude Palm Oil

The option “*Crossbreeding between two species,*” which correctly describes the hybridization process, was selected by 30.12% of answers (Figure 2). Adding the response frequency for “*Oil with two phases (liquid/solid)*” (8.78%), approximately 38.90% of the answers were correct.

However, many participants did not know about hybrid palm oil (26.65%) or provided incorrect answers (34.45%), totaling 61.1%, highlighting the lack of information about the product.

### 3.3. Perception of Hybrid Crude Palm Oil

**Figure 3:**
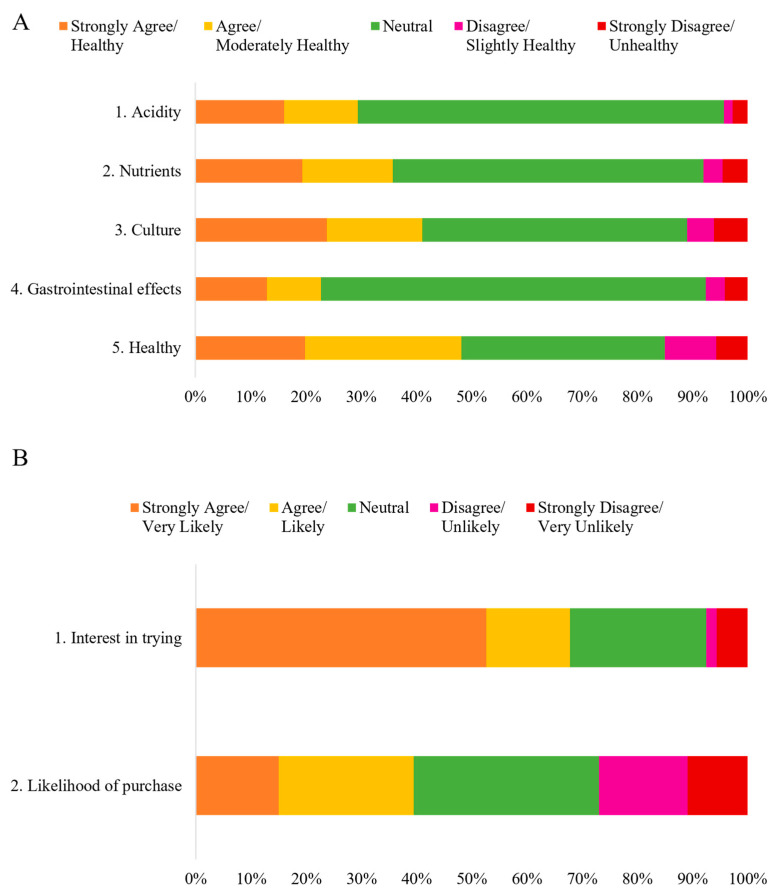
Total number of Brazilian respondents to perception-related questions (**A**): 1. Acidity—The acidity of traditionally sold crude palm oil is perceived to be higher than that of hybrid crude palm oil. 2. Nutrients—Hybrid crude palm oil is perceived to contain more nutrients than traditional crude palm oil. 3. Culture—The popularization and consumption of hybrid crude palm oil are not expected to alter the characteristics of conventional Bahian dishes. 4. Gastrointestinal effects—Hybrid crude palm oil is perceived not to cause gastrointestinal symptoms, such as abdominal pain, upon consumption. 5. Healthy—On a Likert scale from 1 to 5, respondents rated the healthiness of hybrid crude palm oil. (**B**) Potential consumption-related questions: 1. Interest in trying—Respondents indicated their interest in trying hybrid crude palm oil. 2. Likelihood of purchase—On a Likert scale from 1 to 5, respondents rated the possibility of purchasing products made with hybrid crude palm oil (*n* = 1065).

The predominant response across all statements or questions regarding the perception of HCPO was “*Neutral*” (Figure 3A).

**Figure 4:**
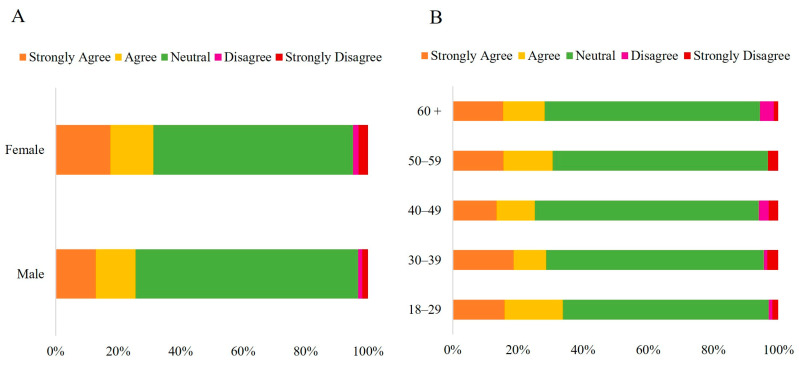
Likert scale response (Strongly Disagree to Strongly Agree) to the statement: “*The acidity of traditionally marketed crude palm oil is higher than that of HCPO*,” categorized by sex (**A**) (*n* = 1060 *) and age group (**B**) (*n* = 1065). Note: * ‘Others’ category under sex (*n* = 5) was excluded from analysis (insufficient cell count for chi-square).

**Figure 5:**
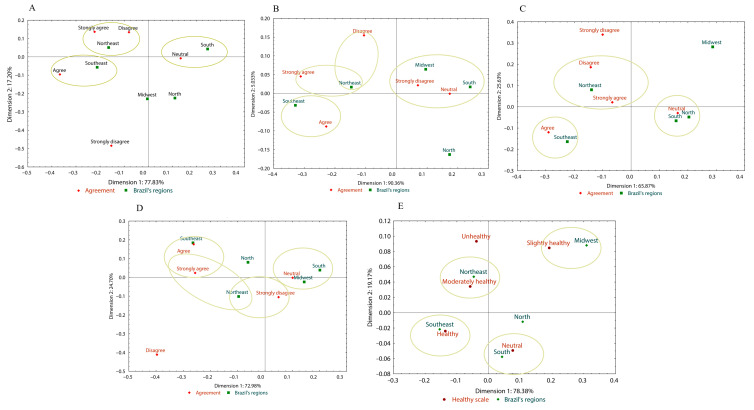
Correspondence analysis of HCPO perceptions regarding acidity (**A**), nutrient content (**B**), culture (**C**), gastrointestinal effects (**D**), and healthiness (**E**) across regions in Brazil, based on survey data.

The results for the first statement, “*The acidity of traditionally marketed CPO is higher than that of HCPO,*” indicate that most participants (66.20%) rated this statement as “*Neutral*” (Figure 4), regardless of sex (*p* = 0.191) and age groups (*p* = 0.122) (Table S1). The correspondence analysis generated two dimensions: the first (Dim. 1), representing 77.83% of the total, and the second (Dim. 2), representing 17.20% and explaining 95.03% of the data (Figure 5).

The South region was associated with the “*Neutral*” option, while the Northeast region was linked to the “*Strongly Agree*” and “*Disagree*” options. The Southeast region was associated with the “*Agree*” option, indicating a firmer tendency in this region to associate HCPO with lower acidity.

Regarding the belief that “*HCPO contains more nutrients than traditional CPO*, “the results suggest that perception varies significantly across different demographic groups, highlighting the importance of considering these factors when communicating information (Table S1).

**Figure 6:**
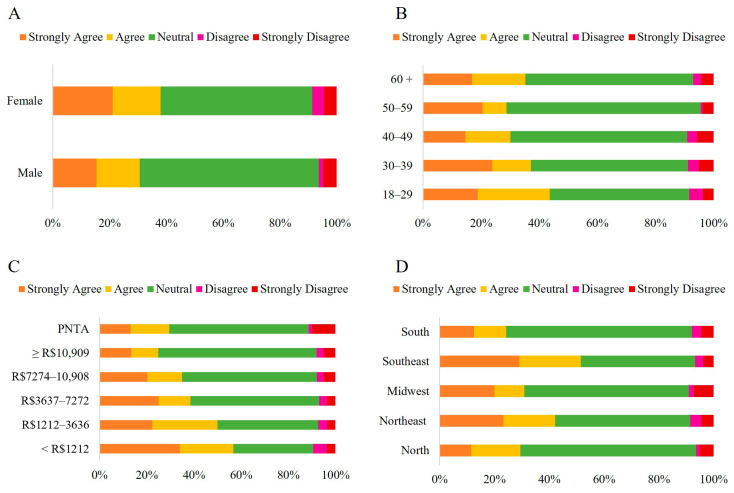
Likert scale response (Strongly Disagree to Strongly Agree) regarding the statement: “*HCPO has more nutrients than conventional crude palm oil,*” categorized by sex (**A**) (*n* = 1060 *); age group (**B**); household income (**C**) and region of residence (**D**) (*n* = 1065). Note: * ‘Others’ category under sex (*n* = 5) was excluded from analysis (insufficient cell count for chi-square); PNTA- Prefer Not to Answer; Brazil’s national minimum salary in 2022 was BRL 1212.00.

The chi-square test revealed a significant difference between males and females (*p* = 0.016) (Table S1). Women showed higher agreement (37.95%, with 21.13% strongly agreeing and 16.82% agreeing) than men (30.60%, with 15.46% strongly agreeing and 15.14% agreeing). Meanwhile, men were more likely to select the “*Neutral*” option (63.09%) than women (53.43%). A significant difference was observed across the age groups (*p* < 0.001). The lowest percentages of “*Neutral*” responses were observed in the 18–29 (48%) and the 30–39 age groups (53.92%), resulting in higher levels of agreement with the statement (43.64% and 37.2%, respectively). The levels of agreement refer to the sum of the “*Strongly Agree*” and “*Agree*” options. The opposite trend was observed in the 50–59 and 40–49 age groups, which had the highest percentages of “*Neutral*” responses (66.88% and 60.90%, respectively) and the lowest levels of agreement to some degree (28.76% and 30.07%, respectively) (Figure 6).

The lowest percentages of “*Neutral*” responses were more prominent in lower-income groups, increasing in higher-income groups (*p* < 0.001). Thus, lower-income groups tended to agree more with the statements than higher-income groups. The participants’ region of residence also affected their responses (*p* < 0.001). The southeastern and northeastern regions showed the highest levels of agreement (51.49% and 41.26%, respectively), whereas other areas demonstrated lower levels of agreement, with the southern region showing the lowest agreement (24.44%). Similarly to the demographic categories above, an inverse relationship was observed between the “*Strongly Agree*” and “*Agree*” options and the “*Neutral*” option. The South region had the highest percentage of “*Neutral*” responses (67.66%), while the Southeast and Northeast regions had the lowest (41.79% and 49.30%, respectively).

The correspondence analysis generated two dimensions: the first (Dim. 1), representing 90.36%, and the second (Dim. 2), representing 5.03%, explaining 95.39% of the data (Figure 5). The South and Midwest regions were associated with the “*Neutral*” and “*Strongly Disagree*” options. In contrast, the Northeast and Southeast regions were linked to the “*Strongly Agree*” and “*Agree*” options, indicating a more positive perception regarding the nutrient content of HCPO. Also, the Northeast region was linked to the “*Disagree*” option.

**Figure 7:**
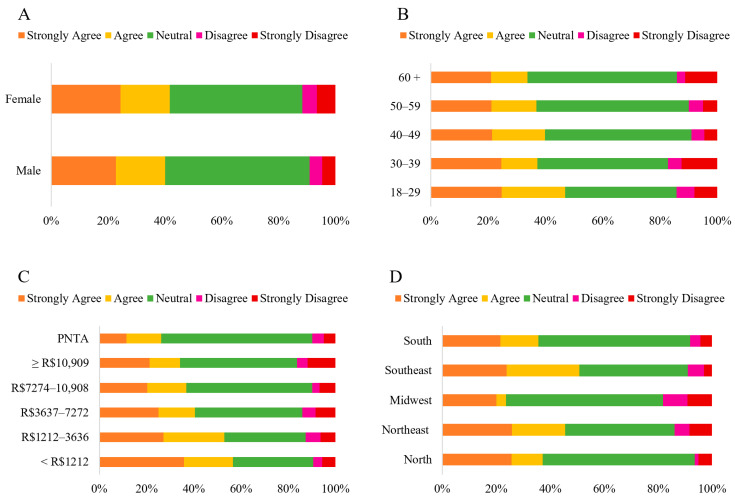
Likert scale response (Strongly Disagree to Strongly Agree) to the statement: “*The popularization and consumption of HCPO will not alter the traditional character of Bahian regional dishes*,” categorized by sex (**A**) (*n* = 1060 *); age group (**B**); household income (**C**) and region of residence (**D**) (*n* = 1065). Note: * ‘Others’ category under sex (*n* = 5) was excluded from analysis (insufficient cell count for chi-square); PNTA- Prefer Not to Answer; Brazil’s national minimum salary in 2022 was BRL 1212.00.

When asked about the possibility of the popularization and consumption of HCPO altering the authenticity of traditional Bahian dishes, the majority perception was “*Neutral*” (47.98%) (Figure 7). The data also indicate that perceptions regarding the preservation of the authenticity of traditional Bahian cuisine due to the popularization and consumption of HCPO vary significantly based on income (*p* = 0.001) and geographic region (*p* < 0.001) (Table S1). It was observed that the higher the income, the greater the percentage of “*Neutral*” responses (33.96% to 53.66%). Conversely, the lower the income, the higher the levels of agreement with the statement (56.60% to 36.81%). Specifically, higher-income categories showed the following agreement percentages: R$7272.00 to R$10,909.00 (36.81%) and above R$10,909.00 (36.91%).

The Southeastern and Northeastern regions had the lowest percentages of “*Neutral*” responses (40.30% and 40.47%, respectively) and the highest percentages of agreement (50.75% and 45.58%, respectively). In contrast, the Midwest region showed the highest rate of “*Neutral*” responses (58.18%) and the lowest levels of agreement (23.64%), as well as the highest percentage levels of disagreement (18.18%). The levels of disagreement refer to the sum of the “*Strongly Disagree*” and “*Disagree*” options.

The correspondence analysis generated two dimensions: the first (Dim. 1), representing 65.87% and the second (Dim. 2), representing 25.63%, explaining 91.50% of the data (Figure 5). The North and South demonstrated neutrality in their opinions. The Southeastern region exhibited a positive perception of the HCPO (Agree). As Bahia is a state in the Northeastern region, where CPO consumption is more traditional than in other regions, this locality was associated with both the “*Disagree*” and “*Strongly Agree*” options, reflecting ambiguity in opinions. Strong cultural attachment to CPO and unfamiliarity with newer HCPO may have contributed to this reaction.

**Figure 8:**
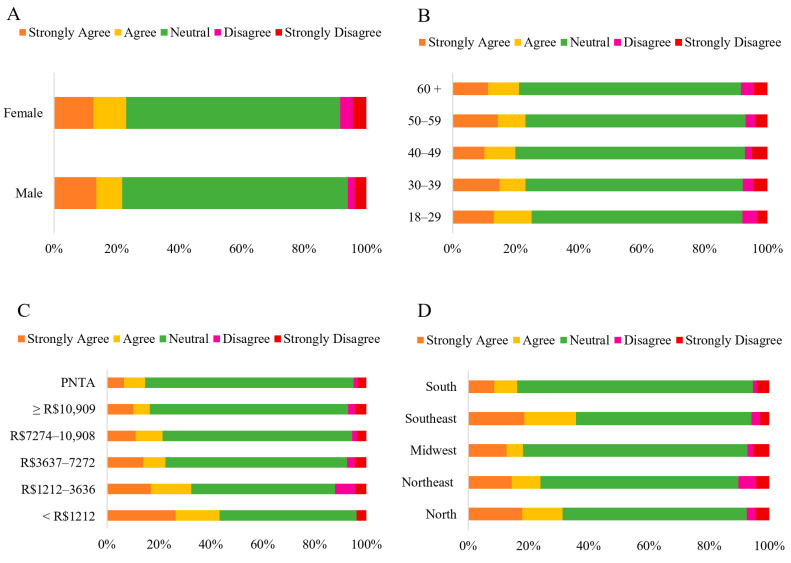
Likert scale response (Strongly Disagree to Strongly Agree) to the statement: “*HCPO does not cause gastrointestinal symptoms such as abdominal pain when consumed*,” categorized by sex (**A**) (*n* = 1060 *); age group (**B**); household income (**C**); and region of residence (**D**) (*n* = 1065). Note: * ‘Others’ category under sex (*n* = 5) was excluded from analysis (insufficient cell count for chi-square); PNTA- Prefer Not to Answer; Brazil’s national minimum salary in 2022 was BRL 1212.00.

Although the majority (69.67%) of participants expressed a “*Neutral*” perception regarding the statement that “*HCPO does not cause gastrointestinal symptoms, such as abdominal pain when consumed*” (Figure 8), significant variations were observed across different demographic groups (Table S2). While no significant differences were found between sex and age groups (*p* > 0.05), income significantly influenced the responses, indicating that participants with lower income levels tended to agree with the statement to some extent (*p* < 0.001) (Figure 5). Geographic region also played a significant role, with participants from the Southeast showing a higher agreement than those from the South (*p* = 0.001).

The correspondence analysis generated two dimensions: the first (Dim. 1), representing 72.98% and the second (Dim. 2), representing 24.70% and explaining 97.68% of the data (Figure 5). The Southeastern region exhibited a positive perception of HCPO (Strongly Agree and Agree), whereas the Northeastern region was associated with both positive and negative perceptions (Strongly Agree and Strongly Disagree).

**Figure 9:**
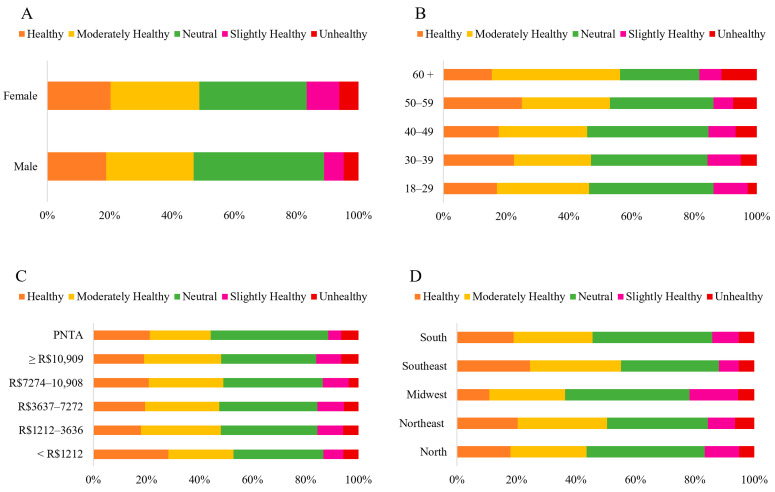
Rating (Unhealthy to Healthy) of the perceived healthiness of HCPO categorized by sex (**A**) (*n* = 1060 *); age group (**B**); household income (**C**); and region of residence (**D**) (*n* = 1065). Note: * ‘Others’ category under sex (*n* = 5) was excluded from analysis (insufficient cell count for chi-square); PNTA- Prefer Not to Answer; Brazil’s national minimum salary in 2022 was BRL 1212.00.

Regarding the perception of “*how healthy do you consider HCPO*,” many participants (36.81%) remained “*Neutral*” (Figure 9). However, when combining the categories “*Healthy*” (19.81%) and “*Moderately Healthy*” (26.36%), it becomes evident that the majority of participants (48.17%) consider this oil to be healthy at some level.

When analyzing sex, income, and region, no significant associations were observed (*p* > 0.05) (Table S2). In contrast, age group showed a significant association (*p* = 0.035), indicating variations in responses based on age. Participants over 60 years old were more likely to classify HCPO as “*Healthy*” or “*Moderately Healthy*” (56.34%), as well as “*Slightly Unhealthy*” or “*Unhealthy*” (18.31%), and were less likely to select “*Neutral*” responses (25.35%). In contrast, the 18–29 and 40–49 age groups were more prone to “*Neutral*” responses (39.64% and 38.72%, respectively) and, consequently, showed lower percentages of classification as “*Healthy*” or “ *Moderately Healthy*” (46.54% and 45.87%, respectively).

The correspondence analysis generated two dimensions: the first (Dim. 1), representing 78.38% and the second (Dim. 2), representing 19.17%, explaining 97.55% of the data (Figure 5). The South and North demonstrated neutrality in their opinions. The Midwestern region was associated with the “*Slightly Unhealthy*” option, while the Northeastern and Southeastern regions were associated with positive perceptions of healthiness (Moderately healthy and Healthy, respectively).

### 3.4. Purchase Potential of Products with Hybrid Crude Palm Oil

**Figure 10:**
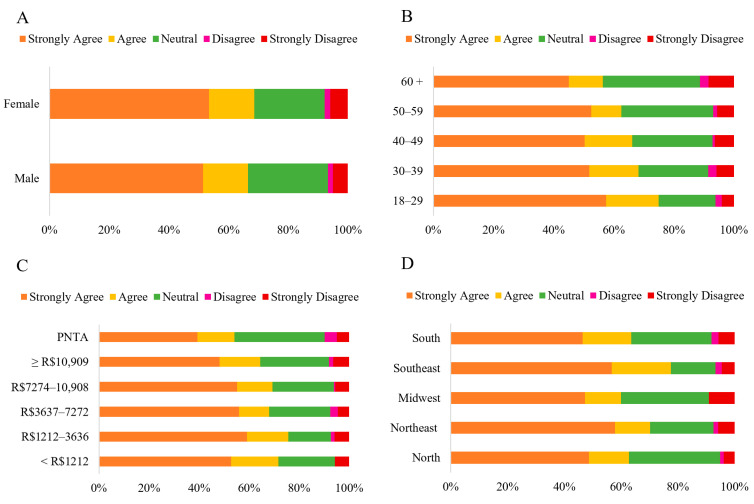
Likert scale response (Strongly Disagree to Strongly Agree) to the statement: “*I am interested in trying HCPO,*” categorized by gender (**A**) (*n* = 1060 *); age group (**B**); household income (**C**) and region of residence (**D**) (*n* = 1065). Note: * ‘Others’ category under sex (*n* = 5) was excluded from analysis (insufficient cell count for chi-square); PNTA- Prefer Not to Answer; Brazil’s national minimum salary in 2022 was BRL 1212.00.

**Figure 11:**
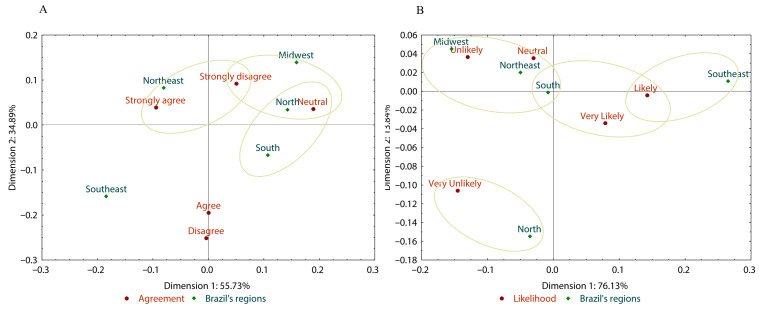
Correspondence analysis between interest in trying HCPO (**A**), likelihood of purchasing foods containing HCPO (**B**) and regions in Brazil, based on survey data.

Two questions were addressed in this category: interest in trying HCPO, and intention to purchase products containing them (Figure 3B). The majority of participants (67.79%) expressed interest in trying HCPO, selecting the “*Strongly Agree*” option (52.58%) and “*Agree*” (15.21%) (Figure 10). The results of the chi-square analysis indicated no significant differences in responses regarding interest in trying HCPO across sex (*p* = 0.818), age groups (*p* = 0.149), income level (*p* = 0.76), and geographic regions (*p* = 0.052) (Table S3). Lieke, Spiller, and Bush (2023) observed that consumers in the Global North prefer foods labeled “*palm oil-free,*” whereas Brazilian consumers demonstrate the opposite perception. The correspondence analysis generated two dimensions: the first (Dim. 1), representing 53.71% and the second (Dim. 2), representing 34.89%, explaining 88.60% of the data (Figure 11).

The Northeast demonstrated agreement regarding trying HCPO (Strongly agree), suggesting a strong interest, possibly due to familiarity with the consumption of CPO, which is deeply rooted in local culinary traditions. However, dissenting responses revealed ambiguous opinions (Strongly disagree). The Midwestern and Northern regions showed strong disagreement or neutrality, indicating resistance or lack of enthusiasm. Meanwhile, the Southern region is associated only with the “*Neutral*” option.

**Figure 12:**
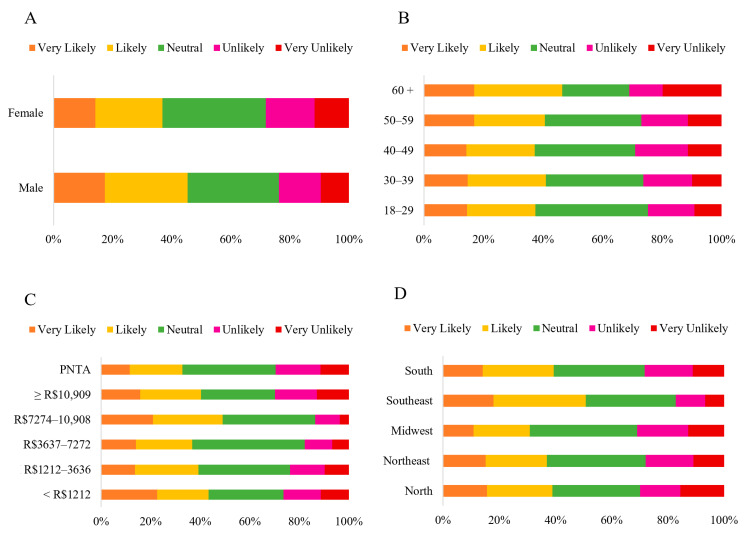
Likelihood of purchasing products containing HCPO, rated on a scale from 1 (None) to 5 (Very High), categorized by sex (**A**) (*n* = 1060 *); age group (**B**); household income (**C**); and region of residence (**D**) (*n* = 1065). Note: * ‘Others’ category under sex (*n* = 5) was excluded from analysis (insufficient cell count for chi-square); PNTA- Prefer Not to Answer; Brazil’s national minimum salary in 2022 was BRL 1212.00.

The majority of participants (39.43%) expressed interest in purchasing products containing HCPO, selecting the options “*Very Likely*” (15.02%) and “*Likely*” (24.41%), compared to the “*Neutral*” option (33.62%) (Figure 12). Therefore, there was a positive predisposition to try the new product, despite the participants’ gaps in knowledge regarding certain nutritional aspects. Responses regarding the likelihood of purchasing HCPO-containing products did not show significant differences across sex (*p* = 0.141), age groups (*p* = 0.576), income levels (*p* = 0.189), and geographic regions (*p* = 0.476) (Table S3).

The correspondence analysis generated two dimensions: the first (Dim. 1), representing 76.13% and the second (Dim. 2), representing 13.84%, explaining 89.97% of the data (Figure 11). The Southeastern region was interested in purchasing HCPO food products (Likely). In contrast, the Midwestern region had a lower interest in buying these products (Unlikely), whereas the Northern region showed no interest in purchasing them (Very unlikely). However, the Northeastern and Southern regions were related to neutral and unlikely opinions. Also, the South region is associated with “*Very Likely*” and “*Likely*” options.

## 4. Discussion

### 4.1. Consumer Profile

The fact that most participants resided in the Northeast region (40.37%) may be correlated with the consumption of crude palm oil in traditional regional dishes such as *acarajé*, *abará*, *vatapá*, and *xinxim de galinha* [25,28]. The wider dissemination of this study within academic circles may explain the predominance of women in the cohort (69.77%). As highlighted by the Brazilian Institute of Geography and Statistics [29], 21.3% of Brazilian women aged 25 years or older have completed higher education, compared to 16.8% of men. This distribution reflects the changes in educational patterns, indicating progress in gender equality in access to education. The concentration in the 30–39 age group suggests significant participation by the millennial generation in the survey. This generation has been recognized for their heightened concerns regarding health and awareness of nutritional issues [30]. Regarding educational level and income, the high percentage of participants with postgraduate degrees, full-time employment, and incomes exceeding nine times the minimum wage suggests a strong presence in the academic community, particularly among educators. The method of dissemination through academic networks and social media may have contributed to this demographic profile.

### 4.2. Knowledge of Hybrid Crude Palm Oil

Oils derived from hybrid plants, produced by crossbreeding different plant species or varieties [1], have emerged as a promising alternative for meeting the growing global demand for vegetable oils [31]. In the case of oil palms, HCPO was developed to provide greater resistance to diseases affecting African palm trees [7]. The *BRS Manicoré*, a Brazilian cultivar, is a hybrid of the African palm *La Mé (Elaeis guineensis Jacq.)* originating from Côte d’Ivoire, and the American palm (*Elaeis oleifera*), native to the Amazon region [7].

Additionally, the proportion of respondents who identified HCPO as a two-phase oil (8.78%) demonstrated an understanding of its physical characteristics, as it presented itself in two fractions, olein (liquid fraction) and stearin (solid fraction), similar to conventional African CPO [6].

The number of incorrect responses highlights the need to clarify the characteristics of HCPO, particularly concerning the crossbreeding process between the two species.

The knowledge gap can be partially explained by the fact that although studies on the hybridization between *Elaeis guineensis* and *Elaeis oleifera* date back to the 1940s, it was only in 2010 that the Brazilian Agricultural Research Corporation (EMBRAPA) introduced the BRS Manicoré cultivar in Brazil [7,23]. In 2009, experiments with the American oil palm began in southern Bahia, specifically in the city of Una, with results published only in 2019 [8,10].

The circulation of information about hybrid palm oil only intensified in 2021, with increased media coverage [32,33]. However, production of HIE OxG in Brazil remains limited. In the North, the Pará state has 11,500 hectares dedicated to this crop, yielding an annual production of 40,000 t [34]. In the Northeast, the Bahia has only 6.12 hectares allocated to this cultivation [8]. These numbers are significantly lower than those of conventional African palm oil production, which is expected to reach 600 Mt in Brazil by 2024 [35].

However, it is important to recognize that disseminating innovative products, such as hybrid palm oil, is a gradual process. Scientific discoveries require time to reach the public. Studies such as these are fundamental for expanding our knowledge and understanding of this oil.

Another portion of the respondents (9.76%) identified oil as being extracted through artisanal methods, which may reflect a gap in information and a lack of awareness regarding industrial extraction methods. This perception may be associated with the traditional artisanal techniques used in Bahia, Brazil, where crude palm oil is produced following ancestral practices of African peoples, such as using a mortar and pestle [36]. However, modern methods in crude palm oil factories involve industrial processes using solvents, steam, water, enzymes, and machinery, including sterilization, stripping, digestion and pressing, clarification, drying, and storage [37].

A minority of the participants (6.48%) mistakenly associated HCPO with transgenic foods, possibly because of a limited understanding of related concepts. This misconception may stem from unfamiliarity with the definition of genetically modified organisms, which, according to the World Health Organization, refers to organisms whose genetic material has been altered in a way that does not occur naturally through mating and/or natural recombination [38].

Additionally, less frequent associations, such as artificial or synthetic oils (1.64%) and light/fit crude palm oil (2.49%), may indicate an incorrect perception of HCPO characteristics. As a relatively new product on the market and with the term “hybrid” in its nomenclature, it is possible that Brazilian consumers interpret the oil as having distinct nutritional or functional qualities. Further clarification of its origin and composition is required.

### 4.3. Perception of Hybrid Crude Palm Oil

Participants were presented with five statements or questions regarding their perception of the HCPO. HCPO has lower acidity than conventional CPO, an intrinsic characteristic of the hybrid fruit, which may enhance its acceptance in Bahian cuisine, widely recognized for regional dishes such as *acarajé*, *moqueca*, and *vatapá* [10,25,28].

The nutritional composition of CPO and HCPO showed notable differences. CPO is predominantly composed of triacylglycerols (95%), with 50% saturated fatty acids (SFA), 40% monounsaturated fatty acids, and 10% polyunsaturated fatty acids, and a significant presence of palmitic acid (44%) and oleic acid (39%) [1]. In contrast, HCPO contains between 52% and 57% oleic acid, 25% to 30% palmitic acid, and up to 13% linoleic acid, along with higher amounts of vitamin E (100 to 2200 mg/kg) and carotenoids (up to 2700 µg/g), indicating a superior nutritional value [11,34].

The perception that HCPO has greater nutritional value than CPO seems to be related to the sociodemographic variables in the present study. Women tended to agree more with this statement, which is consistent with previous evidence of greater attention to nutritional and health information among women [39]. Young adults also showed greater agreement, possibly reflecting a greater familiarity with functional food trends and access to digital information [40]. Regarding income, the differences were significant but not linear, with an emphasis on intermediate ranges, which may combine access to information and more diversified consumption habits. Regional variations highlight that although the Northeast, a region with strong cultural ties to CPO, shows remarkable agreement regarding the nutritional value of HCPO, it is the Southeast that recorded the highest rates of agreement, possibly reflecting less cultural dependence on traditional oil. However, it should be noted that it is not only an oil with nutritional value, but also a cultural element deeply rooted in the Costa do Dendê region of Bahia. This cultural link may influence the perception of nutritional value, especially among groups that value local traditions [41].

In Bahia, CPO, also called “azeite de dendê,” is more than just an ingredient; it is a cultural, religious, and gastronomic symbol associated with African authenticity and central to local cuisine [42]. This cultural and historical connection is reinforced by popular expressions, such as “*do dendê,*” which evoke Bahia and the African diaspora [42]. Given this scenario, the survey data reveal that although almost half of the respondents are neutral, the perception that the popularization of HCPO will not alter the authenticity of Bahian dishes prevails over disagreement (Figure 7). However, this opinion is not homogeneous across social and geographical strata. Significant data (*p* < 0.001) show that lower-income individuals and residents of the Northeast region are considerably more likely to believe in the maintenance of authenticity, indicating that socioeconomic context and cultural proximity are determining factors in this perception, while sex and age do not have a relevant influence. Research on the perception of food authenticity shows that cultural and contextual attributes strongly influence how consumers evaluate traditional products, regardless of innovation in ingredients or production methods [43].

However, the popularity of CPO presents challenges such as negative associations with gastrointestinal symptoms, especially among tourists [32].

It is important to highlight that these perceptions may be influenced by biases towards foods of African origin [44]. Studies indicate that foodborne diseases are frequently associated with traditional Bahian dishes owing to contamination by pathogens in foods such as raw vinaigrette-style salads served alongside *acarajés* [45], as well as *acarajés* [46], *vatapá*, and shrimp [47] sold in Salvador and Belo Horizonte (Brazil). Factors such as improper handling, inadequate sanitation, and prolonged exposure to ambient temperatures may contribute to this issue.

In the present study, although the perception that HCPO does not cause gastrointestinal symptoms was largely neutral, socioeconomic factors such as income and regional (Northeast) were significantly associated with the perceived safety of product consumption (Table S2). This indicates that familiarity with the product, linked to the regional context in the Northeast, particularly Bahia, and socioeconomic factors, plays a relevant role in consumer confidence regarding its safe consumption [48].

Additionally, reported gastrointestinal symptoms may, in some cases, indicate allergic reactions to ingredients such as peanuts, nuts, and shrimp, which are widely used in Bahian cuisine [49,50]. These allergens, which are known to trigger immunoglobulin E (IgE)-mediated responses [51], are more relevant than CPO in the etiology of adverse symptoms.

In contrast, HCPO, rich in oleic acid and bioactive compounds, is considered safe for the recommended daily intake of lipids. Aligned with market trends, such as the “*Clean label*” movement, which emphasizes natural ingredients and transparent labeling, HCPO presents potential for application in new products with functional claims [52,53]. For example [54], nanoencapsulated HCPO is an innovative form of natural antioxidant additive. This approach meets the growing demand for healthier foods free from artificial additives with a lower environmental impact, which distinguishes HCPO as a promising alternative in the vegetable oil market.

### 4.4. Potential for Purchasing Products with Hybrid Crude Palm Oil

Regional differences in the receptivity to HCPO were evident in these data. Higher agreement in the Northeast can be attributed to the strong presence of CPO in traditional dishes [28], while neutrality or disagreement in other regions may be linked to lower traditional consumption of oil or specific regional dietary preferences. Lower sample representation of certain regions may have influenced the results. In the Northeast, openness to consumption suggests potential interest, but resistance from some participants indicates the need for more information about the product.

Food choices are influenced by various factors, including sociocultural, situational, biological, physiological, and psychological aspects, and the intrinsic and extrinsic characteristics of food [52]. Several factors can affect consumers’ purchasing decisions regarding products with nutritional and health claims and crude or refined palm oils. The most common factors include nutritional composition, price, sensory properties (flavor, color, odor, texture, appearance), and sustainability certification [53,55,56,57].

In a study conducted by [25], it was observed that over 50% of respondents indicated a high likelihood of purchasing products containing CPO, whereas more than 30% expressed the same intention regarding refined palm oil. These results reflect the greater predisposition of Brazilian consumers to purchase palm oil products.

In this context, although sociodemographic factors were not significantly associated with the intention to purchase products containing HCPO (*p* > 0.001), some considerations are relevant. Although age was not a determining factor, it might have had an indirect effect. Older individuals tend to cook and purchase oil more frequently, which increases their familiarity and acceptance [58]. This pattern is consistent with studies demonstrating greater culinary involvement among older adults, whereas young adults, due to faster routines and lower culinary skills, more frequently opt for ready-to-eat meals or meals consumed outside the home [59,60]. These studies suggest that purchase decisions may be more strongly associated with behavioral and contextual factors, such as eating habits and product availability, than with isolated demographic variables.

## 5. Conclusions and Future Perspectives

The results of the present study indicate limited knowledge of HCPO among the Brazilian population, with predominantly neutral nutritional and cultural perceptions. Perception is a crucial factor that influences the promotion of HCPO. However, the positive predisposition towards trial and purchase, coupled with its perception as a healthy product, suggests significant potential for market acceptance. The observed regional and demographic disparities are significant, underscoring the urgency of targeted dissemination strategies. To capitalize on this opportunity, future strategies must articulate three main dimensions:

1. Communication and Education: Awareness campaigns should be designed to clearly and technically elucidate the nutritional advantages of HCPO, notably its high oleic acid (ω-9) content and its rich composition of natural antioxidants (carotenoids, vitamin E), which confer significant antioxidant capacity and nutraceutical potential.
2. Sustainable Production Expansion: Overcoming the current scale limitations is imperative. This requires incentives to expand hybrid cultivation in integrated agroforestry systems, which promote socio-biodiversity (e.g., consortia with cocoa) and ensure the environmental and economic sustainability of the chain.
3. Research and Development: Continuous investment in R&D is crucial for advancing the characterization of oil, development of food products, and validation of its beneficial health effects through clinical studies, providing the necessary scientific foundation for its popularization. Equally important is the expansion of these investigations to other Latin American countries, considering aspects of knowledge, perception, and consumption potential, which may foster both population acceptance and the formulation of regional strategies in health, market development, and public policies.

## Figures and Tables

**Table 1:** Questionnaire applied to assess the knowledge, perception, and potential consumption of hybrid crude palm oil.

Area	Questions/Statements	Response Format	Reference
Socioeconomic aspects	Place of residence Sex identification Age group Total number of people in the family Level of education Occupation Family income	Multiple choice (Single answer)	[25]
Knowledge			[26]
	What is meant by the term hybrid crude palm oil?	Multiple choice (Multiple answer)	
Perception			[6,7,10,11,25,26]
	The acidity of traditionally marketed crude palm oil is higher than that of hybrid crude palm oil.	Agree Partially agree Neither agree nor disagree Partially disagree Disagree	
	Hybrid crude palm oil has more nutrients than traditional crude palm oil.		
	The popularization and consumption of hybrid crude palm oil will not alter the authenticity of traditional Bahian dishes.		
	Hybrid crude palm oil does not cause gastrointestinal symptoms such as abdominal pain when consumed.		
	On a Likert scale of 1 to 5, how healthy do you consider hybrid crude palm oil?	Unhealthy Slightly unhealthy Neutral Moderately healthy Healthy	
Potential Consumption			[27]
	I am interested in trying hybrid crude palm oil.	Agree Partially agree Neither agree nor disagree Partially disagree Disagree	
	On a Likert scale of 1 to 5, how likely are you to purchase products with hybrid crude palm oil?	Very Likely Likely Neutral Unlikely Very Unlikely	

**Table 2:** Sociodemographic profile of the participants.

Variable		N	%
Region of residence	Northeast	430	40.38
	South	368	34.56
	Southeast	134	12.58
	North	78	7.32
	Midwest	55	5.16
Sex identification	Female	743	69.77
	Male	317	29.76
	Prefer not to say	3	0.28
	Non-binary	2	0.19
Age group	Between 18 and 29 years	275	25.82
	Between 30 and 39 years	293	27.51
	Between 40 and 49 years	266	24.98
	Between 50 and 59 years	160	15.02
	60 years or older	71	6.67
Number of people living in the household	1	135	12.68
	2	336	31.55
	3	294	27.60
	4	212	19.91
	5	68	6.38
	More than 5	20	1.88
Level of education	Postgraduate	671	63
	Completed undergraduate degree	146	13.71
	Completed high school	233	21.88
	Completed elementary school	8	0.75
	Incomplete elementary school	2	0.19
	Prefer not to answer	5	0.47
Occupation	Full-time/Part-time employment	768	72.11
	Undergraduate/Graduate student	244	22.91
	Not working/Not studying	38	3.57
	Prefer not to answer	15	1.41
Approximate household income	Less than 1 minimum wage	53	4.98
	Between 1 and 3 minimum wages	206	19.34
	Between 3 and 6 minimum wages	200	18.78
	Between 6 and 9 minimum wages	163	15.30
	More than 9 minimum wages	382	35.87
	Prefer not to answer	61	5.73

Reproduced with permission from: [25]. N = number of respondents; % = percentage of responses relative to the total. Note: According to the exchange rate on US$5.045 (https://economia.uol.com.br/cotacoes/cambio/ (accessed on 14 september 2024)), it was equivalent to US$240.219, and Brazil's national minimum salary in 2022 was R$1212.00.

## Data Availability

The original contributions presented in this study are included in the article/Supplementary Material. Further inquiries can be directed to the corresponding author.

## Supplementary Materials

The following supporting information can be downloaded at: https://www.mdpi.com/article/10.3390/foods14183242/s1, Table S1. Association between socio-demographic factors and participant perceptions of Hybrid Crude Palm Oil (HCPO) versus traditional Crude Palm Oil (CPO): A chi-square analysis. Table S2. Association between socio-demographic factors and participant perceptions of the gastrointestinal effects and perceived health value of Hybrid Crude Palm Oil (HCPO): A chi-square analysis. Table S3. Chi-square test of consumption potential among participants in relation to hybrid crude palm oil (HCPO).
