# Supplementary material for: Hybrid Crude Palm Oil in Brazilian Regions: Evaluation of Knowledge, Perceptions, and Consumption Potential
Source: Foods. 2025 Sep 18;14(18):3242. doi: 10.3390/foods14183242 (PMC12469376; doi:10.3390/foods14183242)
Supplement: Supplementary file 1 [file foods-14-03242-s001.zip › foods-3804130-Supplementary S1.pdf]

HYBRID CRUDE PALM OIL IN BRAZILIAN REGIONS: EVALUATION OF KNOWLEDGE, PERCEPTIONS, AND CONSUMPTION POTENTIAL

Agnes Sophia Braga Alves[a]; Deborah Murowaniecki Otero[a, b]; Alana Moreira Bispo[b]; Edilene Ferreira da Silva[c]; Lívia de Matos Santos[a]; Itaciara Larroza Nunes[c]; Maria Cristina Teixeira Cangussu[d]; Cláudio Vaz Di Mambro Ribeiro[a, e]; Camila Duarte Ferreira Ribeiro[a, b*]

[a] Graduate Program in Food Science, School of Pharmacy, Federal University of Bahia, Ondina Campus Salvador, Bahia, 40170-115, Brazil.

[b] Graduate Program in Food, Nutrition, and Health, School of Nutrition, Federal University of Bahia, Canela *Campus*, Salvador, Bahia, 40110-907, Brazil.

[c] Graduate Program in Food Science, Department of Food Science and Technology, Federal University of Santa Catarina, Admar Gonzaga Road, 1346, Itacorubi, Florianópolis, Santa Catarina 88034-000, Brazil.

[d] Graduate Program in Dentistry and Health, School of Dentistry, Federal University of Bahia, 62 - Canela Campus, Salvador, Bahia, 40110-150, Brazil.

[e] School of Veterinary Medicine and Animal Science, Federal University of Bahia, Av. Adhemar de Barros, 500, 40170-110, Salvador, Bahia, Brazil.

*Corresponding author: Nutrition School, Federal University of Bahia, Campus Canela, Salvador, Bahia, 40110-907, Brazil.). E-mail: camiladuartef@ufba.br.

Supplementary Material

Table S1. Association between socio-demographic factors and participant perceptions of Hybrid Crude Palm Oil (HCPO) versus traditional Crude Palm Oil (CPO): A chi-square analysis.

Question	Variables	Strongly Agree		Agree		Neutral		Disagree		Strongly disagree		Total		Chi-square (p-value)
		N	%	N	%	N	%	N	%	N	%	N	%	
The acidity of traditionally marketed crude palm oil is higher than that of hybrid crude palm oil.	**Gender**													0.191
	Masculine	41	12.93	40	12.62	226	71.29	4	1.26	6	1.84	317	100	
	Female	130	17.5	102	13.73	476	64.06	13	1.75	22	2.96	743	100	
	Total	171	16.13	142	13.4	702	66.23	17	1.6	28	2.64	1060*	100	
	**Age**													0.122
	18–29	44	16	49	17.82	174	63.27	3	1.09	5	1.82	275	100	
	30–39	55	18.77	29	9.9	196	66.89	3	1.02	10	3.41	293	100	
	40–49	36	13.53	31	11.65	183	68.8	8	3.01	8	3.01	266	100	
	50–59	25	15.63	24	15	106	66.25	0	0	5	3.13	160	100	
	60 +	11	15.49	9	12.68	47	66.2	3	4.23	1	1.41	71	100	
	Total	171	16.06	142	13.33	706	66.29	17	1.6	29	2.72	1065	100	
**Question**	**Variables**	**Strongly Agree**		**Agree**		**Neutral**		**Disagree**		**Strongly disagree**		**Total**		**Chi-square (p-value)**
		N	%	N	%	N	%	N	%	N	%	N	%	
	**Gender**													0,016
HCPO has more nutrients than CPO.	Masculine	49	15.46	48	15.14	200	63.09	5	1.58	15	4.73	317	100	
	Feminine	15	21.13	125	16.82	397	53.43	31	4.17	33	4.44	743	100	
	Total	20	19.34	174	16.34	597	56.32	36	3.38	49	4.6	1060*	100	

	**Age**													<0.001
	18–29	52	18.91	68	24.73	132	48	13	4.73	10	3.64	275	100	
	30–39	70	23.89	39	13.31	158	53.92	11	3.75	15	5.12	293	100	
	40–49	39	14.66	41	15.41	162	60.9	9	3.38	15	5.64	266	100	
	50–59	33	20.63	13	8.13	107	66.88	1	0.63	6	3.75	160	100	
	60 +	12	16.9	13	18.31	41	57.75	2	2.82	3	4.23	71	100	
	Total	20	19.34	174	16.34	600	56.34	36	3.38	49	4.6	1065	100	
	**Income**													<0.001
	< R$1.212	18	33.96	12	22.64	18	33.96	3	5.66	2	3.77	53	100	
	R$1.212–3.636	46	22.33	57	27.67	88	42.72	8	3.88	7	3.4	206	100	
	R$3.637–7.272	50	25	27	13.5	109	54.5	7	3.5	7	3.5	200	100	
	R$7.274–10.908	33	20.25	24	14.72	93	57.06	5	3.07	8	4.91	163	100	
	≥ R$10.909	51	13.35	44	11.52	256	67.02	12	3.14	19	4.97	382	100	
	PNTA	8	13.11	10	16.39	36	59.02	1	1.64	6	9.84	61	100	
	Total	20	19.34	174	16.34	600	56.34	36	3.38	49	4.6	1065	100	
	**Region**													<0.001
	North	9	11.54	14	17.95	50	60.1	1	1.28	4	5.13	78	100	
	North East	10	23.26	81	18	212	49.3	18	4.19	19	4.42	430	100	
	Midwest	11	20	6	10.96	33	60	1	1.82	4	7.27	78	100	
	Southeast	39	29.1	30	22.39	56	41.79	4	2.99	5	3.73	134	100	
	South	46	12.76	43	11.68	249	67.66	12	3.26	17	4.62	368	100	
	Total	206	19.34	174	16.34	600	56.34	36	3.38	49	4.6	1065	100	

Question	Variables	Strongly Agree		Agree		Neutral		Disagree		Strongly disagree		Total		Chi-square (p-value)
**The popularization and consumption of HCPO will not alter the**		N	%	N	%	N	%	N	%	N	%	N	%	
	**Gender**													0.631

**authenticity of traditional Bahian dishes.**	Masculine	72	22.71	55	17.35	161	50.79	14	4.42	15	4.73	317	100	
	Feminine	181	24.36	129	17.36	346	46.57	38	5.11	49	6.59	743	100	
	Total	253	23.87	184	17.36	507	47.83	52	4.91	64	6.04	1060*	100	
	**Age**													0.07
	18–29	68	24.73	61	22.18	107	38.91	17	6.18	22	8	275	100	
	30–39	79	26.96	40	13.65	146	49.83	15	5.12	40	13.65	293	100	
	40–49	57	21.43	49	18.42	136	51.13	12	4.51	12	4.51	266	100	
	50–59	34	21.25	25	15.63	85	53.13	8	5	8	5	160	100	
	60 +	15	21.13	9	12.68	37	52.11	2	2.82	8	11.27	71	100	
	Total	253	23.76	184	17.28	511	47.98	52	4.88	65	10.89	1065	100	
	**Income**													0.001
	< R$1.212	19	35.85	11	20.75	18	33.96	2	3.77	3	4.92	53	100	
	R$1.212–3.636	56	27.18	53	25.73	71	34.47	13	6.31	13	6.31	206	100	
	R$3.637–7.272	50	25	31	15.5	91	45.5	11	5.5	17	8.5	200	100	
	R$7.274–10.908	33	20.25	27	16.56	87	53.37	5	3.07	11	6.75	163	100	
	≥ R$10.909	88	23.04	53	13.87	205	53.66	18	4.71	49	12.83	382	100	
	PNTA	7	11.48	9	14.75	39	63.93	3	4.92	3	5.66	61	100	
	Total	253	23.76	184	17.28	511	47.98	52	4.88	65	6.1	1065	100	
	**Region**													<0.001
	North	20	25.64	9	11.54	44	56.41	1	1.28	4	5.13	78	100	
	North East	111	25.81	85	19.77	174	40.47	24	5.58	36	8.37	430	100	
	Midwest	11	20	2	3.64	32	58.18	5	9.09	5	9.09	55	100	
	Southeast	32	23.88	36	26.87	54	40.3	8	5.97	4	2.99	134	100	
	South	79	21.47	52	14.13	207	56.25	14	3.8	16	4.35	368	100	
	Total	253	23.76	184	17.28	511	47.98	52	4.88	65	6.1	1065	100	

Note: *In the Chi-square analysis, the total sample size was 1,065. However, for the gender category, an adjusted sample size (N = 1,060) was analyzed, as responses under the non-binary option were excluded due to insufficient numbers across the categories: strongly agree, agree, neutral, disagree, and strongly disagree; PNTA- Prefer Not to Answer; Brazil's national minimum salary in 2022 was BRL 1212.00.

Table S2. Association between socio-demographic factors and participant perceptions of the gastrointestinal effects and perceived health value of Hybrid Crude Palm Oil (HCPO): A chi-square analysis.

Question	Variables	Strongly Agree		Agree		Neutral		Disagree		Strongly disagree		Total		Chi-Square (p-value)
		N	%	N	%	N	%	N	%	N	%	N	%	
**HCPO does not cause gastrointestinal symptoms such as abdominal pain when consumed.**	**Gender**													0.398
	Masculine	43	13.56	26	8.2	229	72.24	7	2.21	12	3.79	317	100	
	Feminine	94	12.65	78	10.5	509	68.51	30	4.04	32	4.31	743	100	
	Total	137	12.92	104	9.81	738	69.62	37	3.49	44	4.15	1060*	100	
	**Age**													0.888
	18–29	36	13.09	33	12	184	66.91	13	4.73	9	3.27	275	100	
	30–39	44	15.02	24	8.19	202	68.94	10	3.41	13	4.44	293	100	
	40–49	27	10.15	26	9.77	194	72.93	6	2.26	13	4.89	266	100	
	50–59	23	14.38	14	8.75	112	70	5	3.13	6	3.75	160	100	
	60 +	8	11.27	7	9.86	50	70.42	3	4.23	3	4.23	71	100	
	Total	138	12.96	104	9.77	742	69.67	37	3.47	44	4.13	1065	100	
	**Income**													<0.001
	< R$1.212	14	26.42	9	16.98	28	52.83	0	0	2	3.77	53	100	
	R$1.212–3.636	35	16.99	32	15.53	114	55.34	16	7.77	9	4.37	206	100	
	R$3.637–7.272	28	14	17	8.5	140	70	6	3	9	4.5	200	100	
	R$7.274–10.908	18	11.04	17	10.43	119	73.01	3	1.84	6	3.68	163	100	
	≥ R$10.909	39	10.21	24	6.28	292	76.44	11	2.88	16	4.19	382	100	
	PNTA	4	6.56	5	8.2	49	80.33	1	1.64	2	3.28	61	100	
	Total	138	12.96	104	9.77	742	69.67	37	3.47	44	4.13	1065	100	
	**Region**													0.001
	North	12	15.35	9	11.54	41	74.55	2	2.56	3	3.85	78	100	

	North East	62	14.42	41	9.53	283	65.81	25	5.81	19	4.42	430	100	
	Midwest	7	12.73	3	5.45	41	74.55	1	1.82	3	5.45	55	100	
	Southeast	25	18.66	23	17.16	78	58.21	4	2.99	4	2.99	134	100	
	South	32	8.7	28	7.61	288	78.26	5	1.36	15	4.08	368	100	
	Total	138	12.96	104	9.77	742	69.67	37	3.47	44	4.13	1065	100	

**Question**	**Variables**	**Healthy**		**Moderately healthy**		**Neutral**		**Slightly healthy**		**Unhealthy**		**Total**		**Chi-square (p-value)**
		N	%	N	%	N	%	N	%	N	%	N	%	
	**Gender**													0.07
	Masculine	60	18.93	89	28.08	133	41.96	20	6.31	15	4.73	317	100	
	Feminine	151	20.32	212	28.53	256	34.45	78	10.5	46	6.19	743	100	
	Total	211	19.91	301	28.4	300	36.7	98	9.25	61	5.75	1060*	100	
	**Age**													0.035
	18–29	47	17.09	81	29.45	109	39.64	30	10.91	8	2.91	275	100	
	30–39	66	22.53	72	24.57	109	37.2	31	10.58	15	5.12	293	100	
	40–49	47	17.67	75	28.2	103	38.72	23	8.65	18	6.77	266	100	
**How healthy do you consider HCPO?**	50–59	40	25	45	28.13	53	33.13	10	6.25	12	7.5	160	100	
	60 +	11	15.49	29	40.85	18	25.35	5	7.04	8	11.27	71	100	
	Total	211	19.81	302	28.36	392	36.81	99	9.3	61	5.73	1065	100	
	**Income**													0.99
	< R$1.212	15	28.3	13	24.53	18	33.96	4	7.55	3	5.66	53	100	
	R$1.212–3.636	37	17.96	62	30.1	75	36.41	20	9.71	12	5.83	206	100	
	R$3.637–7.272	39	19.5	56	28	74	37	20	10	11	5.5	200	100	
	R$7.274– 10.908	34	20.86	46	28.22	61	37.42	16	9.82	6	3.68	163	100	
	≥ R$10.909	73	19.11	111	29.06	137	35.86	36	9.42	25	6.54	382	100	
	NTA	13	21.31	14	22.95	27	44.26	3	4.92	4	6.56	61	100	

Total	211	19.81	302	28.36	392	36.81	99	9.3	61	5.73	1065	100	
**Region**													0.603
North	14	17.95	20	25.64	31	39.74	9	11.54	4	5.13	78	100	
North East	88	20.47	129	30	146	33.95	39	9.07	28	6.51	430	100	
Midwest	6	10.91	14	25.45	23	41.82	9	16.36	3	5.45	55	100	
Southeast	33	24.63	41	30.6	44	32.84	9	6.72	7	5.22	134	100	
South	70	19.02	98	26.63	148	40.22	33	8.97	19	5.16	368	100	
Total	211	19.81	302	28.36	392	36.81	99	9.3	61	5.73	1065	100	

Note: *In the Chi-square analysis, the total sample size was 1,065. However, for the gender category, an adjusted sample size (N = 1,060) was analyzed, as responses under the non-binary option were excluded due to insufficient numbers across the categories: strongly agree, agree, neutral, disagree, and strongly disagree; PNTA- Prefer Not to Answer; Brazil's national minimum salary in 2022 was BRL 1212.00.

Table S3. Chi-square test of consumption potential among participants in relation to hybrid crude palm oil (HCPO).

Question	Variables	Strongly Agree		Agree		Neutral		Disagree		Strongly disagree		Total		Chi-square (p-value)
		N	%	N	%	N	%	N	%	N	%	N	%	
	**Gender**													0.818
	Masculine	163	51.42	48	15.14	85	26.81	5	1.58	16	5.05	317	100	
	Feminine	397	53.43	113	15.21	175	23.55	14	1.88	44	5.92	743	100	
	Total	560	52.58	161	15.19	260	24.53	19	1.79	60	5.66	1060*	100	
	**Age**													0.149
	18–29	158	45.07	48	17.45	52	18.91	6	2.18	11	4	275	100	
	30–39	152	51.88	48	16.38	68	23.21	8	2.73	17	5.8	293	100	
	40–49	134	50.38	42	15.79	71	26.69	2	0.75	17	6.39	266	100	
**I am interested in trying HCPO.**	50–59	84	52.5	16	10	49	30.63	2	1.25	9	5.63	160	100	
	60 +	32	45.07	8	11.27	23	32.39	2	2.82	6	8.45	71	100	
	Total	560	52.58	162	15.21	263	24.69	20	1.88	60	5.63	1065	100	
	**Income**													0.76
	< R$1.212	28	39.34	10	18.87	12	22.64	0	0	3	4.92	53	100	
	R$1.212–3.636	122	59.22	34	16.5	35	16.99	3	1.46	12	5.83	206	100	
	R$3.637–7.272	112	56	24	12	49	24.5	6	3	9	4.5	200	100	
	R$7.274–10.908	90	55.21	23	14.11	40	24.54	1	0.61	9	5.52	163	100	
	≥ R$10.909	184	48.17	62	16.23	105	27.49	7	1.83	24	6.28	382	100	
	PNTA	24	39.34	9	14.75	22	36.07	3	4.92	3	4.92	61	100	
	Total	560	52.58	162	15.21	263	24.69	20	1.88	60	5.63	1065	100	

Question	Variables	Very likely		Likely		Neutral		Unlikely		Very unlikely		Total		Chi-square (p-value)
	**Region**													0.052
	North	38	48.72	11	14.1	25	32.05	1	1.28	3	3.85	78	100	
	North East	249	57.91	53	12.33	96	22.33	7	1.63	25	5.81	430	100	
	Midwest	26	47.27	7	12.73	17	30.91	0	0	5	9.09	55	100	
	Southeast	76	56.72	28	20.9	21	15.67	3	2.24	6	4.48	134	100	
	South	171	46.47	63	17.12	104	28.26	9	2.45	21	5.71	368	100	
	Total	560	52.58	162	15.21	263	24.69	20	1.88	60	5.63	1065	100	

Question	Variables	Very likely		Likely		Neutral		Unlikely		Very unlikely		Total		Chi-square (p-value)
		N	%	N	%	N	%	N	%	N	%	N	%	
**How likely are you to purchase products with HCPO?**														0.141
	Masculine	55	17.35	89	28.08	98	30.91	45	14.2	30	9.46	317	100	
	Feminine	105	14.13	169	22.75	260	34.99	123	16.55	86	11.57	743	100	
	Total	160	15.09	258	24.34	358	33.77	168	15.85	116	10.94	1060*	100	
	**Age**													0.576
	18–29	40	14.55	63	22.91	104	37.82	43	15.64	25	2.91	275	100	
	30–39	43	14.68	77	26.28	96	32.76	48	16.38	29	9.9	293	100	
	40–49	38	14.29	61	22.93	90	33.83	47	17.67	30	1.28	266	100	
	50–59	27	16.88	38	23.75	52	32.5	25	15.63	18	1.25	160	100	
	60 +	12	16.9	21	29.58	16	22.54	8	11.27	14	19.72	71	100	
	Total	160	15.02	260	24.41	358	33.62	171	16.06	116	10.89	1065	100	
	**Income**													0.189
	< R$1.212	12	22.64	11	20.75	16	30.19	8	15.09	6	6.11	53	100	
	R$1.212–3.636	28	13.59	53	25.75	76	36.89	29	14.08	20	9.71	206	100	
	R$3.637–7.272	23	14.11	37	22.7	74	45.4	18	11.04	11	6.75	200	100	

R$7.274– 10.908	34	20.86	46	28.22	61	37.42	16	9..2	6	3.68	163	100	
≥ R$10.909	60	15.71	94	24.61	114	29.84	65	17.02	49	12.83	382	100	
PNTA	7	11.48	13	21.31	23	37.7	11	18.03	7	11.48	61	100	
Total	160	15.02	260	24.41	358	33.62	171	16.06	116	10.89	1065	100	
**Region**													0.476
North	13	16.67	18	23.08	24	30.77	11	14.1	12	15.38	78	100	
North East	65	15.12	94	21.86	151	35.12	73	16.98	47	10.93	430	100	
Midwest	6	10.91	11	20	21	38.18	10	18.18	7	12.73	55	100	
Southeast	24	17.91	44	32.84	43	32.09	14	10.45	9	6.2	134	100	
South	52	14.13	93	25.27	119	32.34	63	17.12	41	11.14	368	100	
Total	160	15.02	260	24.41	358	33.62	171	16.06	116	10.89	1065	100	

Note: *In the Chi-square analysis, the total sample size was 1,065. However, for the gender category, an adjusted sample size (N = 1,060) was analyzed, as responses under the non-binary option were excluded due to insufficient numbers across the categories: strongly agree, agree, neutral, disagree, and strongly disagree; PNTA- Prefer Not to Answer; Brazil's national minimum salary in 2022 was BRL 1212.00.
