# Supplementary material for: Hybrid Crude Palm Oil in Brazilian Regions: Evaluation of Knowledge, Perceptions, and Consumption Potential
Source: Foods. 2025 Sep 18;14(18):3242. doi: 10.3390/foods14183242 (PMC12469376; doi:10.3390/foods14183242)
Supplement: Supplementary file 1 [file foods-14-03242-s001.zip › foods-3804130-Supplementary S2.pdf]

HYBRID CRUDE PALM OIL IN BRAZILIAN REGIONS: EVALUATION OF KNOWLEDGE, PERCEPTIONS, AND CONSUMPTION POTENTIAL

Agnes Sophia Braga Alves[a]; Deborah Murowaniecki Otero[a, b]; Alana Moreira Bispo[b]; Edilene Ferreira da Silva[c]; Lívia de Matos Santos[a]; Itaciara Larroza Nunes[c]; Maria Cristina Teixeira Cangussu[d]; Cláudio Vaz Di Mambro Ribeiro[a, e]; Camila Duarte Ferreira Ribeiro[a, b*]

[a] Graduate Program in Food Science, School of Pharmacy, Federal University of Bahia, Ondina Campus Salvador, Bahia, 40170-115, Brazil.

[b] Graduate Program in Food, Nutrition, and Health, School of Nutrition, Federal University of Bahia, Canela *Campus,* Salvador, Bahia, 40110-907, Brazil.

[c] Graduate Program in Food Science, Department of Food Science and Technology, Federal University of Santa Catarina, Admar Gonzaga Road, 1346, Itacorubi, Florianópolis, Santa Catarina 88034-000, Brazil.

[d] Graduate Program in Dentistry and Health, School of Dentistry, Federal University of Bahia, 62 - Canela Campus, Salvador, Bahia, 40110-150, Brazil.

[e] School of Veterinary Medicine and Animal Science, Federal University of Bahia, Av. Adhemar de Barros, 500, 40170-110, Salvador, Bahia, Brazil.

*Corresponding author: Nutrition School, Federal University of Bahia, Campus Canela, Salvador, Bahia, 40110-907, Brazil.). E-mail: camiladuartef@ufba.br.

Supplementary S2- Questions

Socioeconomic Questions

1. What is your age group?
2. Which state do you live in?
3. What gender do you identify with?
4. What is your level of education?
5. What is your current occupation?
6. How many people live in your household, including yourself?
7. Considering your income along with that of the person(s) living with you, what is the approximate household income?

Hybrid Crude Palm Oil Questions

1- What do you understand hybrid crude palm oil to be?

( ) Crossbreeding between two species
( ) Genetically modified food
( ) Blend of two different oils
( ) Artisanally extracted oil
( ) Oil and water mixture
( ) Oil with two phses (liquid/solid)
( ) Artificial/Synthetic oil
( ) Light/Fit palm oil
( ) I don't know the answer

WHAT DO YOU THINK ABOUT HYBRID PALM OIL?

2- Indicate your agreement with the questions below about hybrid palm oil:

1- Strongly agree; 2- Agree; 3 – Neutral; 4- Disagree; 5- Strongly disagree

	1	2	3	4	5
The acidity of traditionally marketed crude palm oil is higher than that of hybrid crude palm oil.					
Hybrid crude palm oil has more nutrients than traditional crude palm oil.					
The popularization and consumption of hybrid crude palm oil will not alter the authenticity of traditional Bahian dishes.					
Hybrid crude palm oil does not cause gastrointestinal symptoms such as abdominal pain when consumed.					
I am interested in trying hybrid crude palm oil.					

2- On a Linkert scale of 1 to 5, how healthy do you consider hybrid palm oil?

1- Unhealthy; 2- Slightly healthy; 3- Neutral; 4- Moderately healthy; 5-Healthy

1	2	3	4	5

3- On a Linkert scale of 1 to 5, how likely are you to purchase products with hybrid crude palm oil?

1- Very unlikely; 2- Unlikely; 3- Neutral; 4- Likely; 5- Very Likely

1	2	3	4	5
